# Dataset on the dynamic structural parameters of an old existing building exposed to earthquake loading before and after strengthening techniques – A case study, Lebanon

**DOI:** 10.1016/j.dib.2018.11.077

**Published:** 2018-11-20

**Authors:** Moustafa Moffed Kassem, Fadzli Mohamed Nazri

**Affiliations:** School of Civil Engineering, Universiti Sains Malaysia, Engineering Campus, 14300 Nibong Tebal, Penang, Malaysia

## Abstract

This paper provides data and information on Beirut Arab University׳s existing old building in Lebanon. The building was primarily designed to resist gravity loads only, with no attention to seismic or lateral load effects. The data shows that there is a need to improve the conditions of the existing old building by applying new features that function as seismic capacity resistance. The first feature was applied by adding RC-shear walls to the existing building, and the second feature was by implementing concentric steel bracing at the peripheral face of the building. In this article, a set of critical parameters to identify the seismic design of buildings was associated in this data. The parameters were described in terms of dynamic properties as follows: (a) natural fundamental period, (b) mode shapes, (c) torsional irregularity, (d) stiffness and ductility, and (e) plastic hinges formation. Particularly, the estimated cost analysis of added materials plays an important role in choosing the suitable retrofitting technique for old buildings.

**Specifications table**

**Table:** 

Subject area	*Civil Engineering*
More specific subject area	*Structural Engineering, Earthquake Engineering*
Type of data	*Table, figure, graph*
How data was acquired	*Desktop computer, Simulation platform, Survey and field data; Soil Laboratory report was done by the university.*
Data format	*Analysed*
Experimental factors	*The data were obtained from the school of civil engineering is as follow: (1) Structural configuration layout of the existing building, (2) Building location on the seismic map zone in (z = 0.25 g), (3) The non-destructive test report that estimate the concrete strength, (4) Simulation under lateral load pattern, (5) Soil test report data.*
Experimental features	*The existing building and its alternative retrofitting techniques were modelled using ETABS software and S-concrete Software.*
Data source location	*Data collected from Beirut, Beirut Arab University, were analysed in the Computer Lab in the School of Civil Engineering Campus, Universiti Sains Malaysia, 14300 Nibong Tebal, Penang, Malaysia.*
Data accessibility	*Data are presented in this article.*
Related research article	–

**Value of the data**

• Data can be used to keep alerting the specialists, researchers, professionals and governmental bodies for the important of the seismic rehabilitation due to high risk of earthquakes in Beirut-Lebanon.
• Data can be used to guide the designers, seismologists, and construction industries to the concepts of placing, designing, and execution of new alternative retrofitting materials to the old existing building which is very necessary in seismic design, especially in terms of bonding and anchoring with old elements.
• Data can be used for researchers who deals with modeling softwares and computational approaches in strengthening and retrofitting of old buildings in order to be satisfied with the earthquake resistant design of the structures.
• Data can be extremely valuable for most earthquake-engineering researchers to understand the role of the dynamic properties on the behaviour of the structural building under the effect of earthquake loading. For examples, the natural fundamental period, mode shapes, torsional irregularity, stiffness, and ductility.
• Data can be employed in choosing the most appropriate technique to be considered in retrofitting Beirut Arab University through the cost estimation or feasibility study which is necessary aspect for all engineers and contractors in any particular projects.

## 1 Data

A huge percentage of Reinforced Concrete (RC)-buildings in Beirut were built and set-up without the essential earthquake layout design requirements, therefore, these buildings are considered the most vulnerable to earthquakes. The building, which is an old structure, was primarily designed to resist gravity loads only, without giving any attention to the lateral load.

The main purposes of this data article were to evaluate the seismic performance of an RC-building in Beirut under seismic loading, and to propose a solution for the structural rehabilitation and strengthening of the existing building using shear walls and steel bracing, and to estimate the material cost of each retrofitting technique. The data related to seismic performance of the existing building (Model 1) was measured and compared with two other retrofitting techniques: Model 2 (with added shear walls) and Model 3 (with added concentric steel bracing) based on the dynamic properties.

**Fig. 1:**
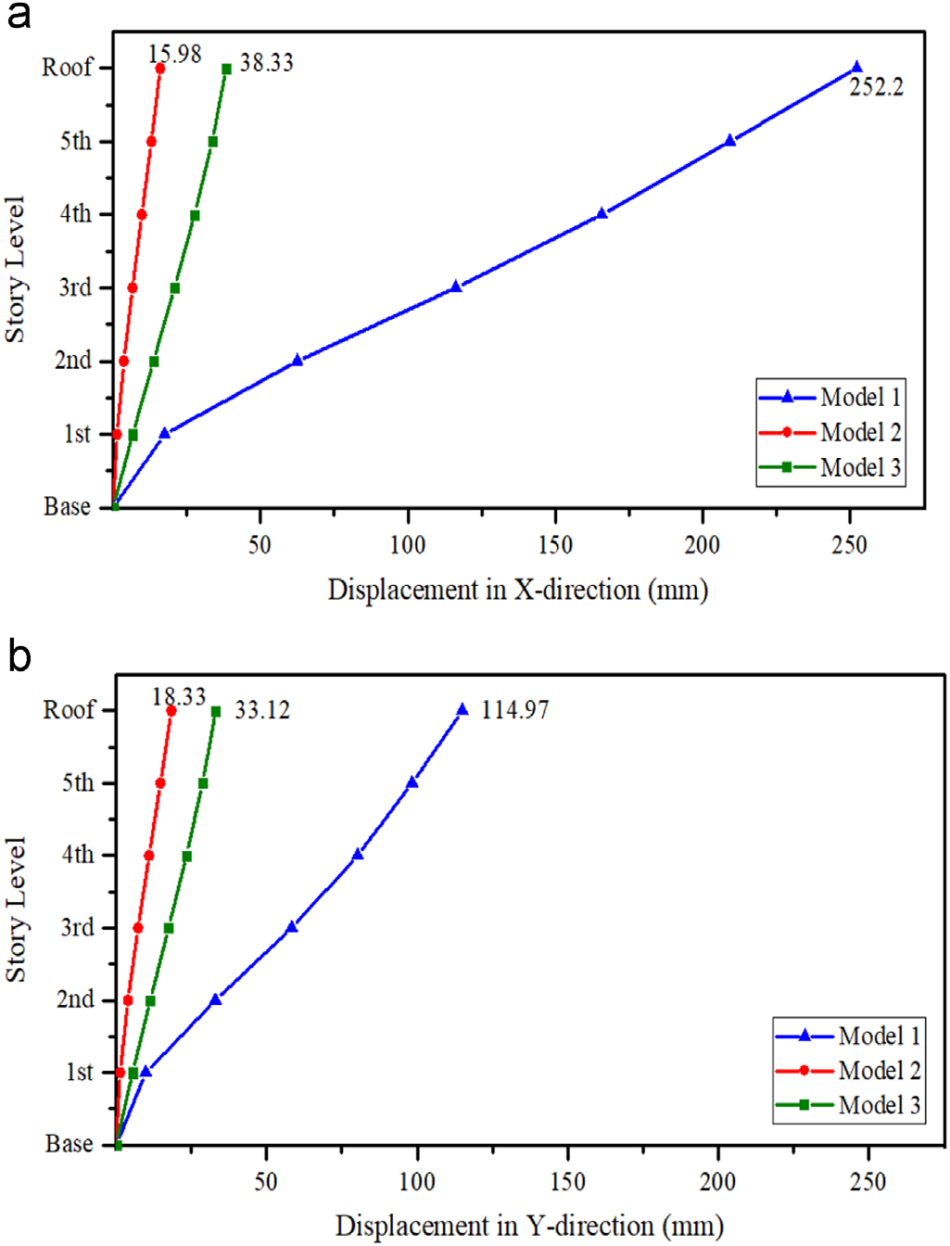
Maximum in-elastic displacement, (a) *X*-direction and (b) *Y*-direction.

**Fig. 2:**
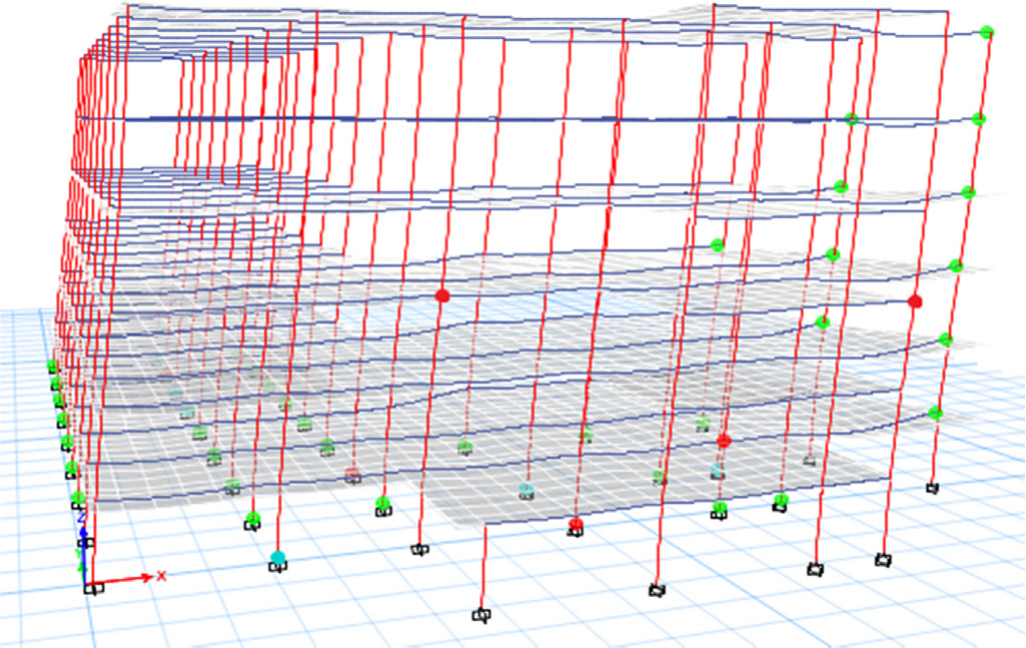
Formation of plastic hinges in Model 1 (existing building).

**Fig. 3:**
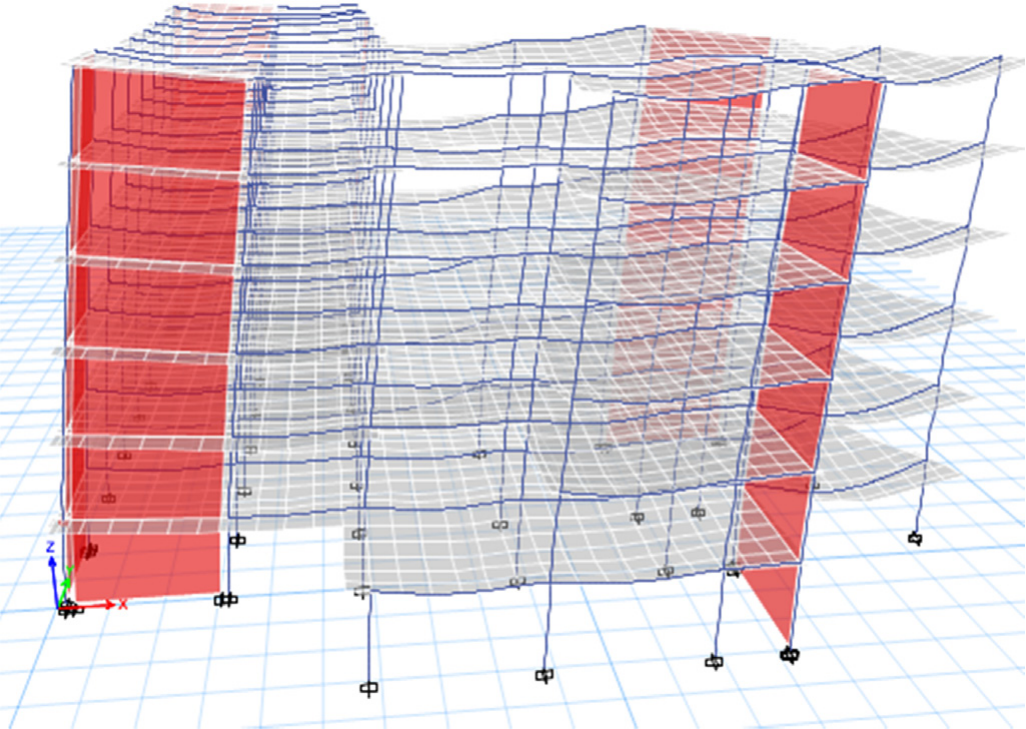
No formation of plastic hinges in Model 2 (retrofitted with shear walls).

**Fig. 4:**
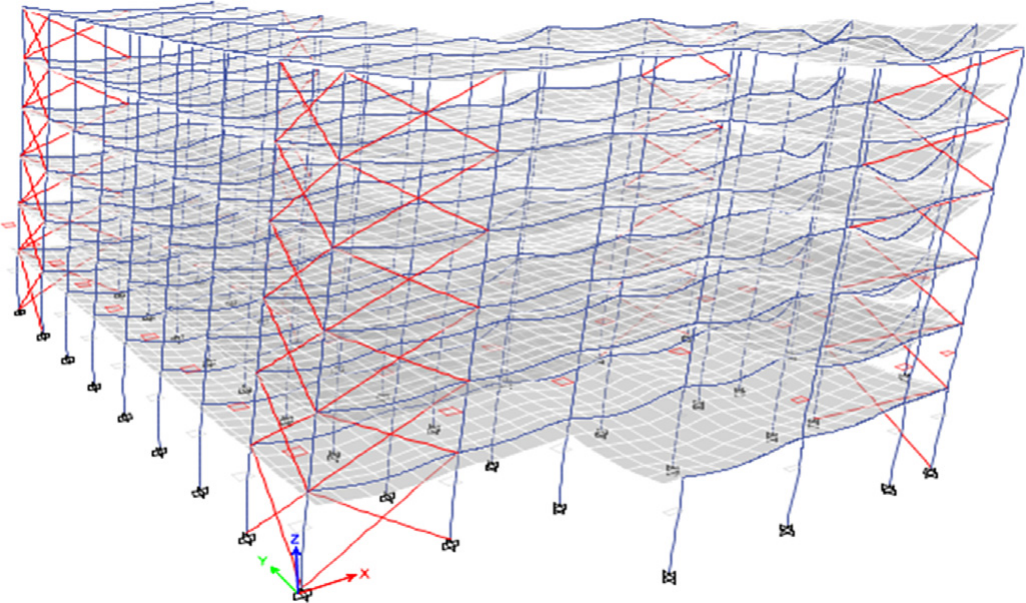
No formation of plastic hinges in Model 3 (retrofitted with concentric steel bracing).

**Fig. 5:**
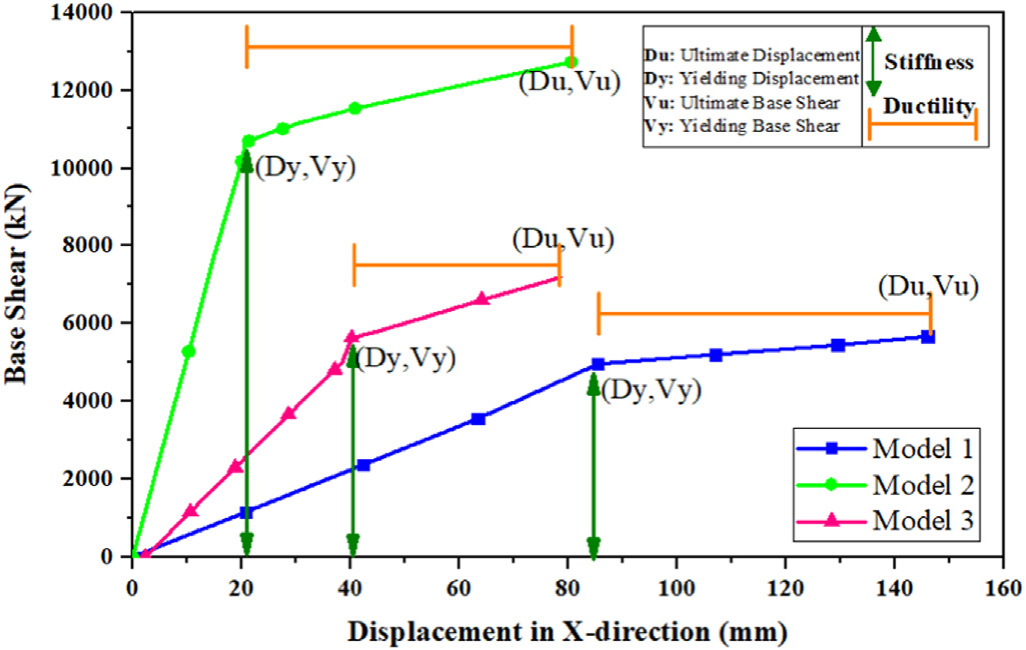
Pushover curves for existing and strengthened models.

**Fig. 6:**
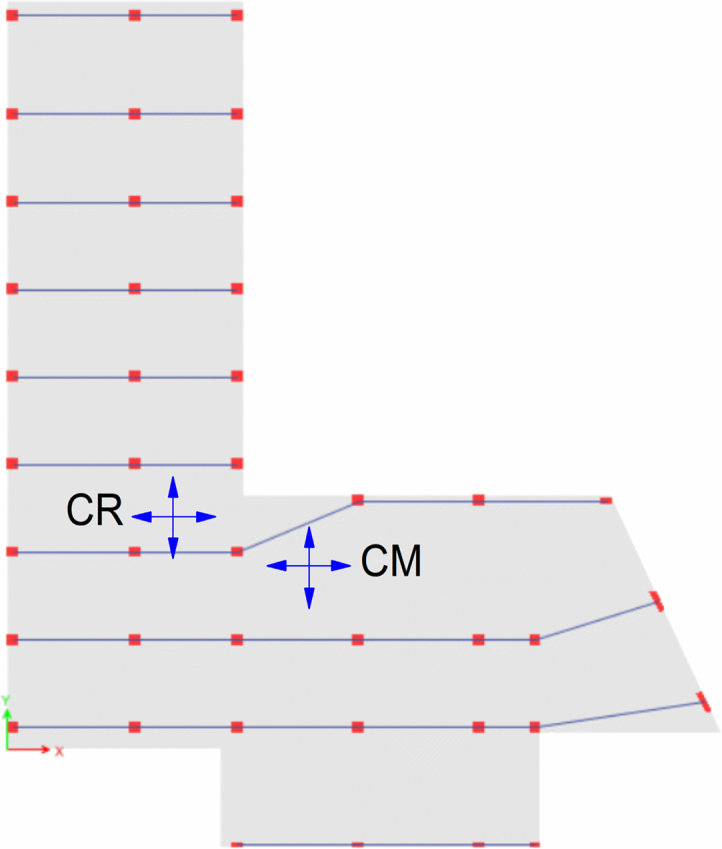
Location of CM and CR of the existing building.

**Fig. 7:**
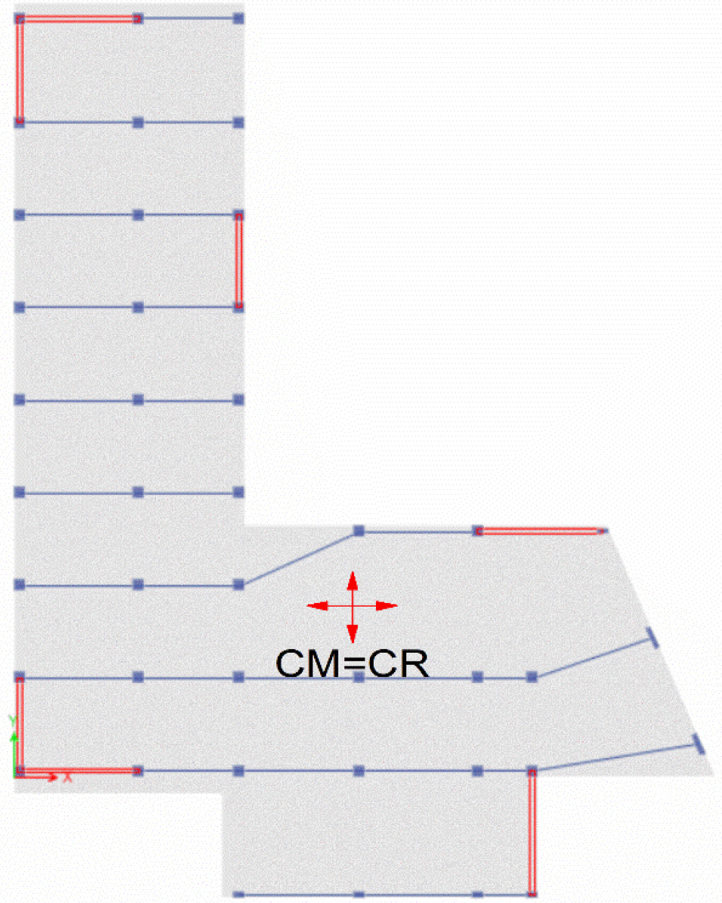
Location of CM and CR of Model 2.

**Fig. 8:**
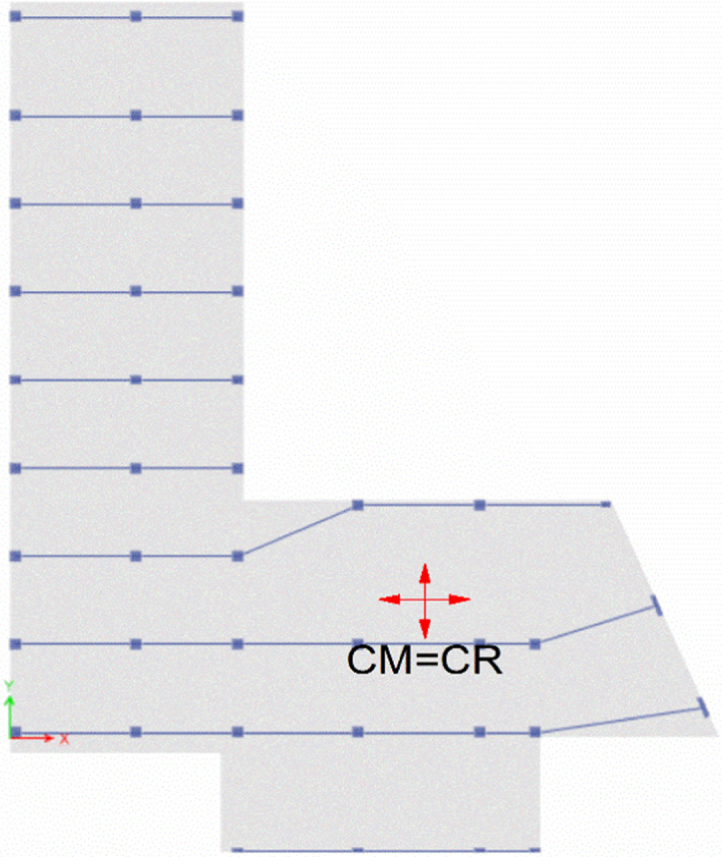
Location of CM and CR of Model 3.

The data were generated based on the following dynamic properties: (1) maximum displacement, (2) natural fundamental period, (3) mode shapes, (4) torsional irregularity in terms of centre of mass and centre of rigidity, (5) stiffness and ductility using pushover analysis, (6) plastic hinges formation, and the estimated material costs as illustrated in Fig. 1, Fig. 2, Fig. 3, Fig. 4, Fig. 5, Fig. 6, Fig. 7, Fig. 8, and in Table 1, Table 2, Table 3, Table 4, Table 5, Table 6, Tablee 7, Table 8, Tablee 9, Table 10.

### 1.1 Inelastic displacement

See Fig. 1.

### 1.2 Mode shapes, and period

See Table 1, Table 2, Table 3.

**Table 1:** Three-dimensional mode shapes and period of the existing building (Model 1).

**Table 2:** Three-dimensional mode shapes and period of Model 2 (with added shear walls).

**Table 3:** Three-dimensional mode shapes and period of Model 3 (with added steel bracing).

### 1.3 Plastic hinges formation

See Fig. 2, Fig. 3, Fig. 4.

### 1.4 Stiffness and ductility using pushover analysis

See Fig. 5.

### 1.5 Torsional irregularity with Centre of Mass (CM) and Centre of Rigidity (CR) location

See Table 4, Table 5, Table 6 and Fig. 6, Fig. 7, Fig. 8.

**Table 4:** Torsional irregularity ratio of the existing building in *X* and *Y* directions.

Story	Load Case	Max Drift	Average Drift	Ratio	Status
Story6	EQX	0.007791	0.004149	**1.878**	Inadequate
	EQY	0.004507	0.002337	**1.928**	
Story5	EQX	0.008311	0.004437	**1.873**	Inadequate
	EQY	0.005763	0.003088	**1.867**	
Story4	EQX	0.008459	0.004515	**1.873**	Inadequate
	EQY	0.006921	0.003795	**1.824**	
Story3	EQX	0.009143	0.004873	**1.876**	Inadequate
	EQY	0.007315	0.004093	**1.787**	
Story2	EQX	0.008944	0.004759	**1.879**	Inadequate
	EQY	0.006071	0.003477	**1.746**	
Story1	EQX	0.004202	0.00224	**1.876**	Inadequate
	EQY	0.002546	0.001492	**1.706**	

^*^EQX: Earthquake in *X*-direction, EQY: Earthquake in *Y*-direction.

^*^ Inadequate: Torsion Exist.

**Table 5:** Torsional irregularity ratio of Model 2 (retrofitted with shear walls in *X* and *Y* directions).

Story	Load Case	Max Drift	Average Drift	Ratio	Status
Story6	EQX	0.00164	0.0015	**1.091**	Adequate
	EQY	0.002202	0.001922	**1.14**	
Story5	EQX	0.00173	0.00159	**1.088**	Adequate
	EQY	0.002308	0.00196	**1.177**	
Story4	EQX	0.00172	0.00159	**1.085**	Adequate
	EQY	0.002182	0.001887	**1.157**	
Story3	EQX	0.00156	0.00145	**1.082**	Adequate
	EQY	0.001881	0.00166	**1.133**	
Story2	EQX	0.0012	0.00111	**1.079**	Adequate
	EQY	0.001368	0.001239	**1.104**	
Story1	EQX	0.00059	0.00054	**1.075**	Adequate
	EQY	0.000632	0.000595	**1.062**	

^*^EQX: Earthquake in *X*-direction, EQY: Earthquake in *Y*-direction.

^*^Adequate: Torsion does not exist.

**Table 6:** Torsional irregularity ratio of Model 3 (retrofitted with steel bracing in *X* and Y directions).

Story	Load case	Max drift	Average drift	Ratio	Status
Story6	EQX	0.00299	0.00263	**1.137**	Adequate
	EQY	0.001286	0.001144	**1.125**	
Story5	EQX	0.00401	0.00345	**1.161**	Adequate
	EQY	0.001556	0.001385	**1.123**	
Story4	EQX	0.00465	0.00398	**1.168**	Adequate
	EQY	0.001725	0.001536	**1.123**	
Story3	EQX	0.00498	0.00426	**1.17**	Adequate
	EQY	0.001779	0.001586	**1.122**	
Story2	EQX	0.00517	0.00437	**1.181**	Adequate
	EQY	0.001702	0.001526	**1.116**	
Story1	EQX	0.00384	0.00326	**1.17**	Adequate
	EQY	0.001118	0.001028	**1.088**	

^*^EQX: Earthquake in X-direction, EQY: Earthquake in *Y*-direction.

^*^Adequate: Torsion does not exist.

### 1.6 Material cost estimation data

The cost estimation data for the proposed strengthening schemes was performed based on the current prices (2017) report that was published by BLOMINVEST BANK in Lebanon (See Tablee 7, Table 8, Tablee 9, Table 10).

**Tablee 7:** Cost estimation for steel bracing system material.

Cost estimation for Steel Bracing System (USD)	
Cross-Sectional Area for Bracing (m^2^)	0.007536
Mass (kg)	399.4683
Rate/kg (USD)	**2.5**
Total Number of Braces	84
Total Cost (USD)	**83,888**

**Table 8:** Cost estimation for shear wall system concrete material.

Cost estimation for Shear Wall System concrete material (USD)	
Walls thickness (cm)	25
Walls Length (m)	36.6
Walls Height (m)	21
Rate/m^2^ (USD)	**100**
Total Cost (USD)	**76,860**

**Tablee 9:** Cost estimation for the reinforcement of the shear wall system.

Cost estimation for Shear Wall System steel reinforcement (USD)			
Reinforcing Bars	**T16**	**T14**	**T12**
Kg/m	1.58	1.21	0.89
Rebar Length	12 m		
Number of bars	1421	90	1765
Total Mass(ton)	26.6	1	18.63
Rate/ton (USD)	**483**		
Total Cost (USD)	**22,330**

**Table 10:** Total cost evaluation for the two-retrofitting system.

Total Cost Evaluation for the two retrofitting systems (USD)	
Shear Wall System (Model 2)	Steel Bracing (Model 3)
99,190	**83,888**

## 2 Experimental design, materials, and methods

### 2.1 Data flow

The computational approach provided in this data article is to check the validity of the existing old building structure under seismic loading and to develop the dynamic properties using ETABS software as a simulation platform tool; firstly, Model 1(existing building) should only be analysed for gravity loads, then, re-modelled to assess its capacity and performance when seismic forces are applied. Secondly, by analysing Model 2 (retrofitted with shear walls system) as a rehabilitation technique by applying seismic forces. Finally, by analysing Model 3 (retrofitted with X-steel bracing system) using the same seismic forces. The seismic loading protocol used was based on the seismic zone (Lebanon) in accordance with UBC1997 code and Lebanon seismic norm. In this case study, the dynamic characteristic was necessary to be considered in the structural modeling of BAU׳s main building. Nevertheless, earthquake engineering mostly focuses on structural properties by analysing and determining a) natural fundamental period for cracked sections, b) number of mode shapes, c) torsion irregularity, d) drift, and e) stiffness and ductility under seismic loading.

The experimental design, materials and method of this data is described as follow:

1. Data collection-Building description.
2. Soil and Foundation data report of the existing building.
3. Evaluated Materials data report of the existing building (Non-destructive test).
4. Computational modeling approach using ETABS (Model 1, Model 2, and Model 3).
5. Retrofitting design and execution concept based on FEMA-273.
6. Modeling the Evaluated Material.
7. Design the models based on the standard code specifications (ACI-318, UBC1997).
8. Beam and column stiffness modifier.
9. Apply the nonlinear static analysis (POA)-lateral load pattern.
10. Generating the dynamic properties (In-elastic displacement, Number of mode shapes, torsion irregularity, stiffness, and ductility).

**Fig. 9:**
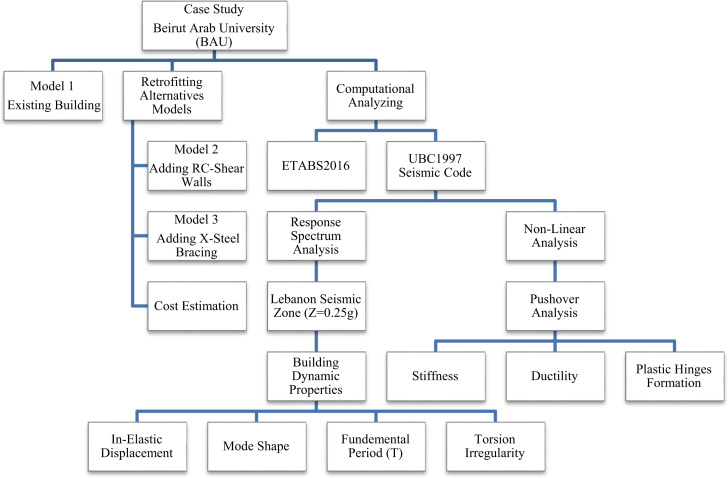
Data and methodology flowchart.

The flow chart in Fig. 9 describes and summarises the data flow and the methodology of work for the case study at Beirut Arab University (BAU).

### 2.2 Data collection: building description

Structural building description of Beirut Arab University (BAU) is described in Table 11.

**Table 11:** Structural building description.

Beirut Arab University main building structural characteristics	
Building Typology	Reinforced concrete building
Structural System Type	Beam-Column system (Framing system)
Total height	21 m
Number of stories	6
Story height	3.5
Column dimension	120 × 30 cm^2^, 60 × 60 cm^2^, 30 × 60 cm^2^
Type of Beams	Drop beams, Embedded beams
Type of Slab, and Thickness	Ribbed Slab, 30 cm
Foundation Type	Isolated footing and Spread Footing

### 2.3 Soil and foundation data report

Based on the geotechnical assessment performed on the soil/foundation system done by the university, the following properties were derived:

a. The site consisted of reddish brown with fine sand with loose to medium density and the absence of water.
b. Standard Penetration Test (SPT) was performed at a different location on the site to find the bearing capacity (B.C = 3 kg/cm^2^) of the soil below the footings.
c. The SPT test was mostly done to find the bearing capacity of soil. Meyerhof (1956) suggested the net allowable bearing pressure with the SPT –*N* number of blows values that correspond to 2.54 cm as an allowable settlement. Eqs. (1) and (2) were used to find the net allowable bearing capacity (*q_net,all_*) when the width of the footing (B) was less than 1.22 m, or more than 1.22 m respectively.
  (1) $$q_{net,\;all}=11.98N\;\mathrm{for}\;B\;\leq 1.22m\;\left(\mathrm{Mpa}\right)$$
  (2) $$q_{net,\;all}=7.99N{\left(\frac{3.28B+1}{3.28B}\right)}^{2}\;\mathrm{for}\;B\;\geq 1.22m\;\left(\mathrm{Mpa}\right)$$

**Table:** 

Depth (m)	SPT (N) Number of Blows	Unit Weight *γ* (ton/m^3^)	Angle of friction, *φ* (deg)	Cohesion, c (tons/m^2^)

0–3	11	1.6	33	0
4–10	21	1.7	35	0
d. Soil Parameters

### 2.4 Evaluated materials data report

The quality of the structural materials was considered in a methodology of work, relying on a set of core tests and non-destructive tests to evaluate the concrete strength. The procedure included an examination of the size and location of the reinforcing bar. Based on the data in the test report done by the university, the materials used in the existing building are as follows:

1. **Reinforcing steel rebars**

– The use of cover metre survey at different locations constitutes a general overview of the reinforcement properties. It shows a cover thickness between from 3 to 7 cm and generally, 5 cm is taken as the average value.
– The bars used have a diameter 16 mm (T16 bars) in the columns and the ribbed slabs.
– A Plain reinforcing bar made of mild steel has a yielding stress fy = 260 MPa.

2. **Concrete compressive strength**
  The compressive strength of concrete is generally in the range of 20–30 Mpa, due to a set of non-destructive tests that were done to evaluate the concrete strength, a minimum of 20 MPa is used for conservative purposes. Table 12 shows the concrete compressive strengths of the four samples.

**Table 12:** Concrete compressive strengths.

Sample number	Compressive strength (MPa)
Sample 1	23.8
Sample 2	22.7
Sample 3	24.6
Sample 4	28.4

### 2.5 Computational modelling approach

**Fig. 10:**
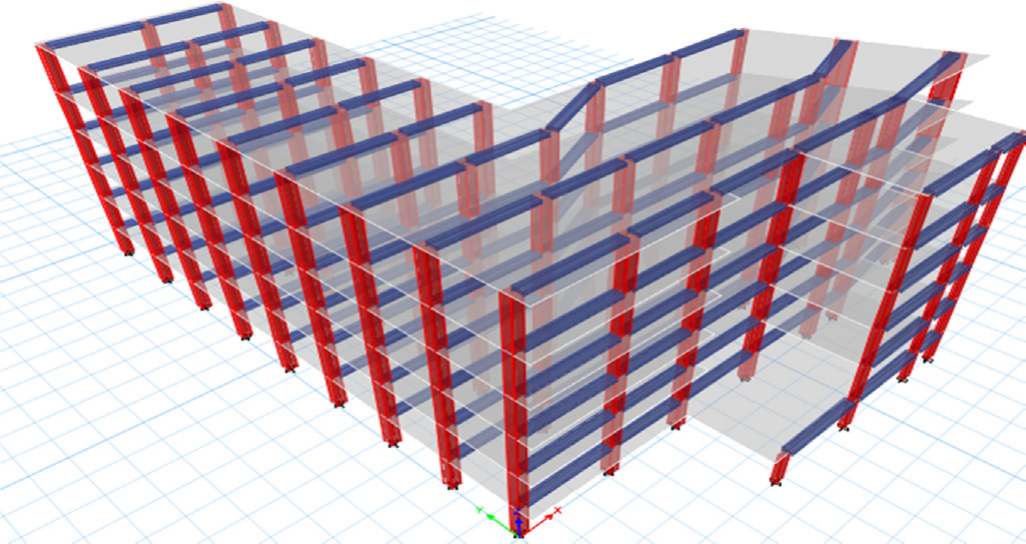
Existing building (Model 1).

**Fig. 11:**
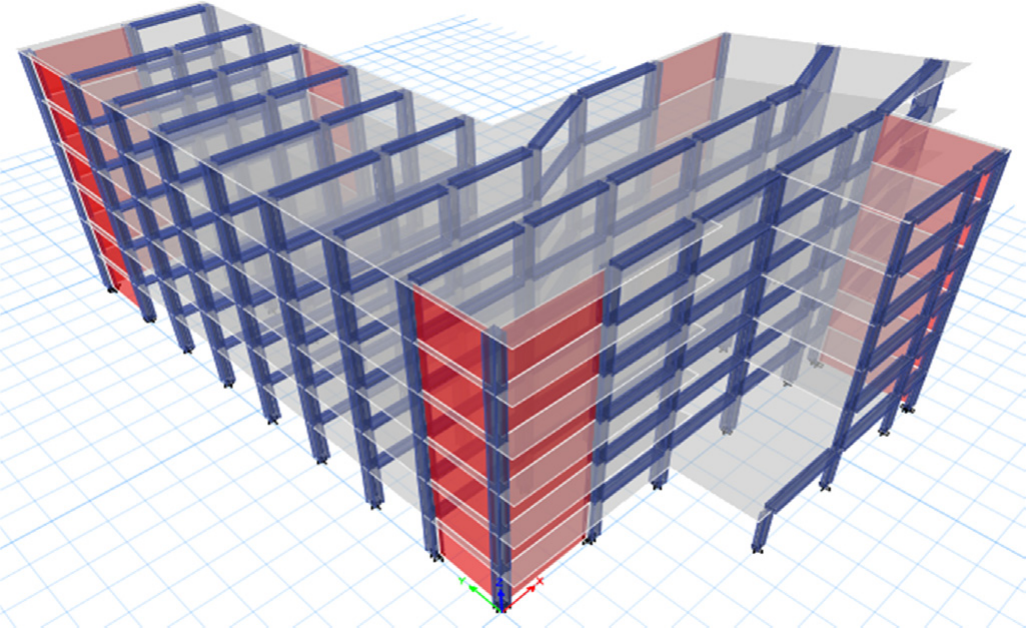
Retrofitted by adding shear walls (Model 2).

**Fig. 12:**
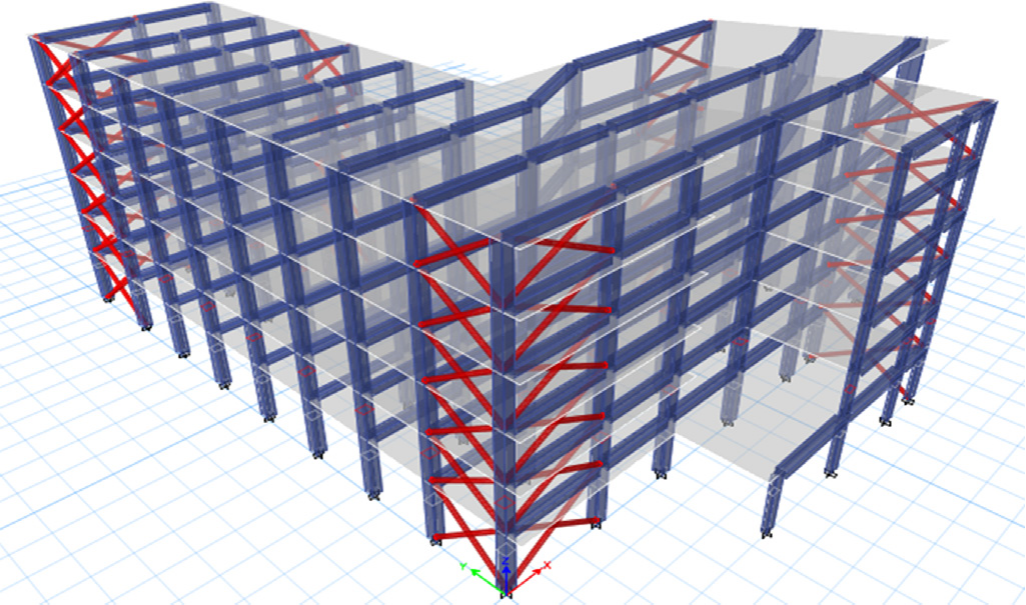
Retrofitted by adding concentric steel bracing (Model 3).

ETABS software was used to generate a 3-D model before proceeding with the analysis. The following models were performed and shown in Fig. 10, Fig. 11, Fig. 12:

1. The case study was analysed under the effect of gravity loads only.
2. The case study was analysed under the effect of seismic and gravity loads (Model 1).
3. The case study was analysed with the addition of shear walls (Model 2).
4. The case study was analysed with the addition of concentric steel bracing (Model 3).

#### 2.5.1 Retrofitting design and execution concept

For shear walls, the design and execution concept were based on the following criteria:

1. The columns must serve as boundary member of the shear walls, and well-confined end regions of RC walls are necessary for terms of the ductility of the wall [1]. In addition, reinforcement continuity to the wall is considerably important to achieve adequate moment capacity as shown in Fig. 13.

**Fig. 13:**
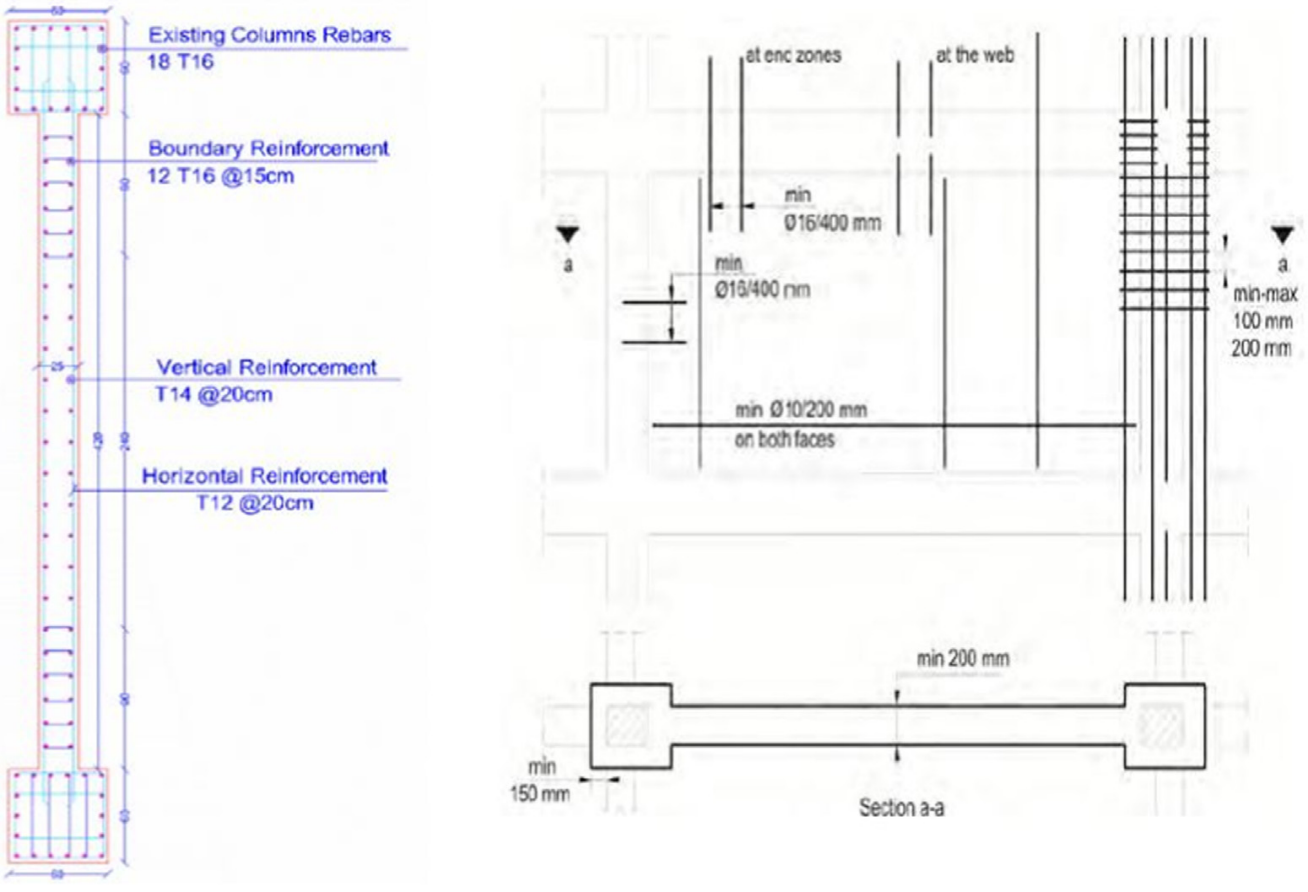
Shear wall detailing with column connection and end region boundaries.
2. The new walls must be placed on the exterior edge of the building to avoid torsion by ensuring the center of mass and the center of rigidity are close to each other and irregularities of the structure are eliminated.
3. The bonding between the old and the new elements majorly affects the function of the desired resistance system. Anchors between the elements and special treatments for connecting surfaces are highly required in the shear wall addition alternative.
4. The Federal Emergency Management Agency 547 [2], provides some valuable and practical techniques for execution the seismic retrofitting methods of the existing building as shown in Fig. 14.

**Fig. 14:**
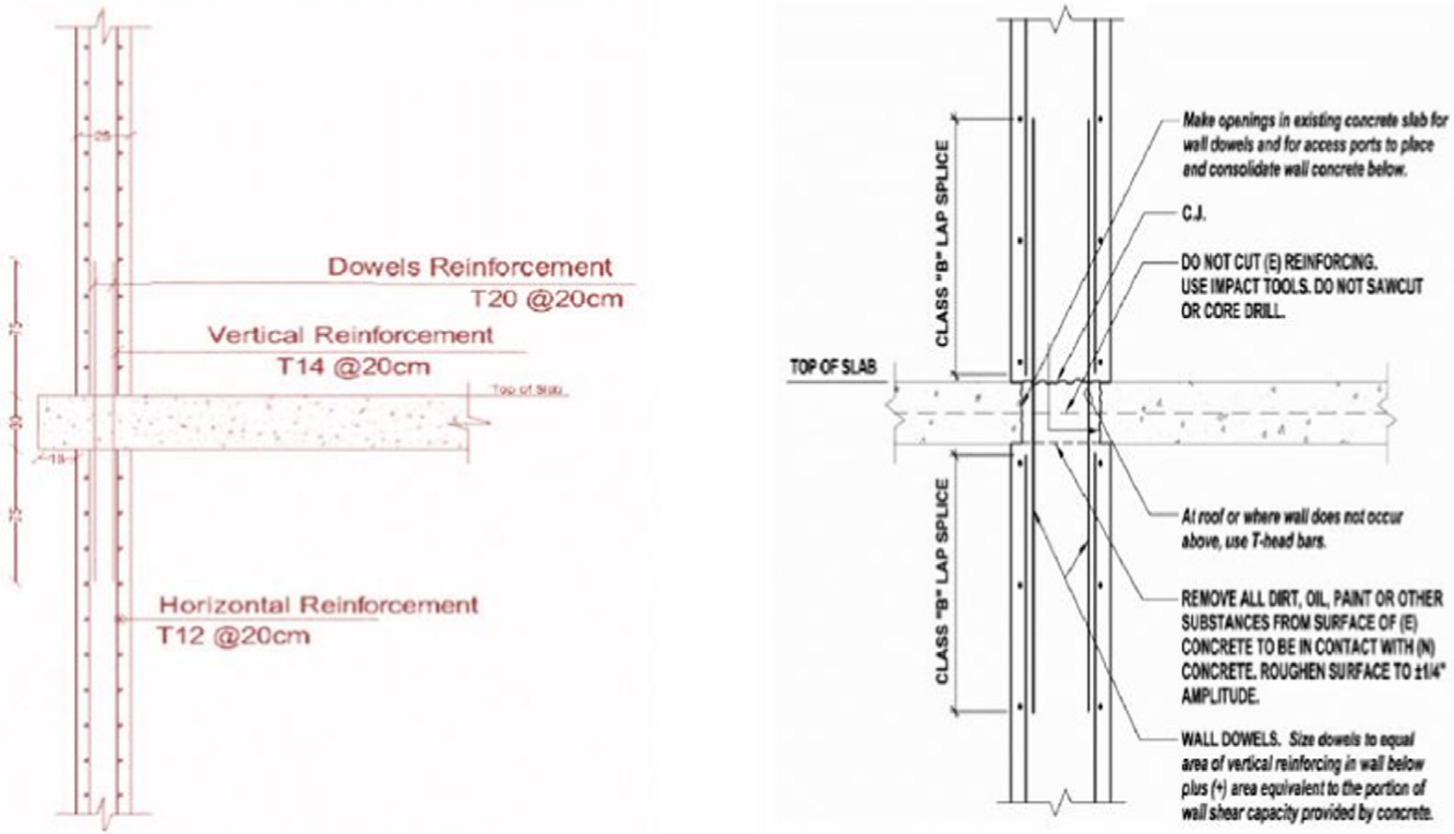
Shear wall detailing with slab connection.

For steel bracing, the design and the execution concept were based on the following features:

1. The concentric X-braces must be placed in the same location as the shear walls, which is along the edges of the building.
2. Braces must be pin connected to the column-beam joint through welding to gusset plates connections as shown in Fig. 15(a) and (b).

**Fig. 15:**
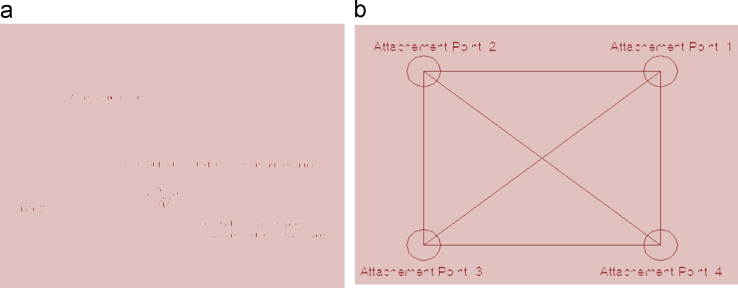
(a) Detailed section of the steel brace to RC elements connection, (b) Steel bracing attached to the concrete element at 4 points.
3. The moment interaction check will govern the design of the braces. If the sum of the ratios is less than one (PMM ratio < 1.0) the design of the brace with a diameter of 25 cm and thickness of 1 cm is adequate as can be seen in Table 13 and Fig. 16.

**Table 13:** Design capacity of the added steel bracing.

Section	Moment interaction check (PMM)	Status
Brace 25 × 1 cm^2^	0.407	Passed
Brace 25 × 1 cm^2^	0.395	Passed
Brace 25 × 1 cm^2^	0.705	Passed
Brace 25 × 1 cm^2^	0.726	Passed
Brace 25 × 1 cm^2^	0.455	Passed
Brace 25 × 1 cm^2^	0441	Passed
Brace 25 × 1 cm^2^	0.629	Passed
Brace 25 × 1 cm^2^	0.499	Passed
Brace 25 × 1 cm^2^	0.464	Passed
Brace 25 × 1 cm^2^	0.476	Passed
Brace 25 × 1 cm^2^	0.743	Passed
Brace 25 × 1 cm^2^	0.517	Passed
Brace 25 × 1 cm^2^	0.394	Passed
Brace 25 × 1 cm^2^	0.469	Passed
Brace 25 × 1 cm^2^	0.644	Passed
Brace 25 × 1 cm^2^	0.867	Passed
Brace 25 × 1 cm^2^	0.513	Passed
Brace 25 × 1 cm^2^	0.645	Passed

**Fig. 16:**
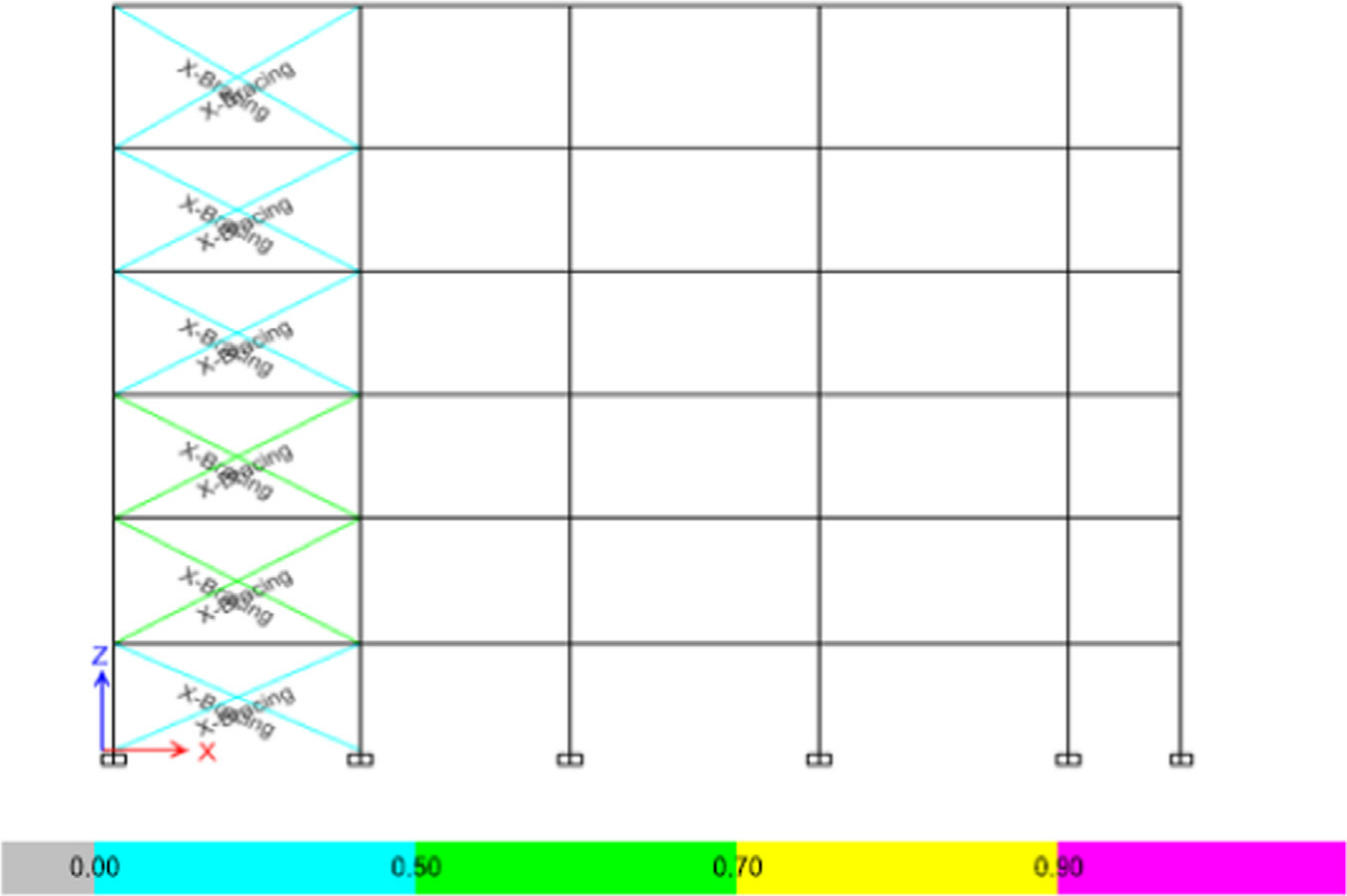
Steel bracing moment interaction capacity in the elevation view.

#### 2.5.2 Modelling the evaluated material

The compressive strength of the existing columns and beams was based on the non-destructive tests. On the other hand, the compressive strength of the new walls was modelled to be more than that of the original building, which was equal to 30 MPa. Moreover, the concrete modulus of elasticity was frequently expressed in terms of compressive strength. Table 14 represents the compressive strength and modulus of elasticity of the material used. The following empirical equation Eq. (3) was used to compute the modulus of elasticity:

(3) $$\mathrm{Ec}=4700\sqrt{f^{\prime }c}\;\left(MPA\right)$$

**Table 14:** Compressive strength and modulus of elasticity of concrete.

Structural elements	Compressive strength (MPa)	Modulus of elasticity (MPa)
Existing Columns	20	21,019
Existing Beams, Slabs	20	21,019
New Walls	30	25,742

Similarly, the yield strength of the steel reinforcing bars (rebars) of the original building was related to the cover metre survey test mentioned above, while for the new walls, the yield strength of the rebars was modelled to be more than that of the original building, which was equal to 500 MPa. Table 15 shows the yield strength and modulus of elasticity of the rebars. Furthermore, the X-steel bracing section used in this study is illustrated in Table 16.

**Table 15:** Yield strength and modulus of elasticity of rebars.

Structural elements	Yielding strength (MPa)	Modulus of elasticity (MPa)
Existing	260	200,000
New System (Shear Walls)	500	200,000

**Table 16:** Dimension and type of X-steel bracing section.

Steel bracing section	Type of bracing
Hollow circular pipe, Diameter 250 mm, and thickness 10 mm	X-Bracing

#### 2.5.3 Code specifications

The Standard NL135:2012 (Lebanese seismic norm) considers the French and American codes, in terms of structural seismic engineering, that present in Lebanon [3]. It gives some flexibilities by allowing designers to use either the French Norm or the American codes and UBC97 when building their structures [4], [5], [6]. The “Building Code Requirements for Structural Concrete” was used in this study. It covers the materials, design, and construction of the structural concrete used in building structures. The Code also covers the strength evaluation of existing concrete structures [7].

#### 2.5.4 Beam and column stiffness modifier

Beams and columns were modeled as frame elements, while the slab and walls as shell elements. The stiffness modifiers were used to consider the structural elements as cracked sections instead of gross sections. Stiffness modifiers have a significant effect on the behavior of the structure, where in the absence of modifiers, a structure would be stiff. Thus, overestimating stiffness can be a negative point in some cases especially under the effect of lateral loads. The modifier factors are related to the building code ACI as tabulated in Table 17.

**Table 17:** Stiffness modifier factors of the structural elements [7].

Structural Elements	ACI (318-14)	ETABS
Beam	0.35Ig	I_22_ = I_33_ = 0.35
Column	0.7Ig	I_22_ = I_33_ =0.70
Wall	0.7Ig	f_11_ = f_22_ = 0.70
Slab	0.25Ig	m_11_ = m_22_ = 0.25

#### 2.5.5 Design load cases

All the three structural model loads (Model 1, Model 2, and Model 3) were assigned according to different parameters and imposed to lateral, dead, and live loads. The lateral loads were designed based on the UBC97 code. As for the dead loads, the parameters were the self-weight of the structure, weight of the partition such as finishes, brick wall and all permanent constructions called superimposed dead load, in addition, to live load based on ASCE code. Table 18 shows the uniform distributed loads considered as the dead and live load. The seismic load parameter data related to Lebanon zone with 0.25 g ground acceleration are tabulated in Table 19. The presence of gravity loads and lateral loads must be considered in the combinations of actions for design. Then, from the UBC97 under clause 1612.2, the combinations of both seismic and gravity actions Eqs. (4), (5) and (6) are:

**Table 18:** Uniform distributed gravity loads [8].

Gravity load	All floors (KPa)
Super Imposed Dead Load (SIDL)	5
Live Load	5

**Table 19:** Seismic load parameter data related to Lebanon zone [3].

Lebanon seismic zone parameters	
Soil profile	Sc
Seismic zone factor	*Z* = 0.25
Seismic acceleration coefficients	0.29
Seismic velocity coefficients	0.4

For gravity load ultimate strength design (*U*):

(4) U=1.2DL+1.6LL

For seismic and gravity loads ultimate strength design (*U*):

(5) U=1.2DL+1.0EQX(Envelope)

(6) U=1.2DL+1.0EQY(Envelope)

The terms EQX and EQY are the seismic load cases in the X and Y-directions, respectively, and the envelope condition is to provides the maximum response and generate over-designed under extreme loading.

#### 2.5.6 Design of columns

Code ACI318-14 was used to design the concrete column frame sections of the existing building. Compressive strength (f ׳c) of the concrete was 20 MPa^,^ and the yield stress of reinforcement steel was 260 MPa. Table 20 shows the column section size and reinforcement steel details.

**Table 20:** Column section size and reinforcement steel details.

Case Study	Column size and reinforcement		
	Column 60 × 60 cm^2^	Column 60 × 30 cm^2^	Column 120 × 30 cm^2^
Existing Building			
Reinforcement bars	Vertical: 18T16	Vertical: 12T16	Vertical: 18T16
	Horizontal: T10@150	Horizontal: T10@150	Horizontal: T10@150

#### 2.5.7 Torsional irregularity

**Fig. 17:**
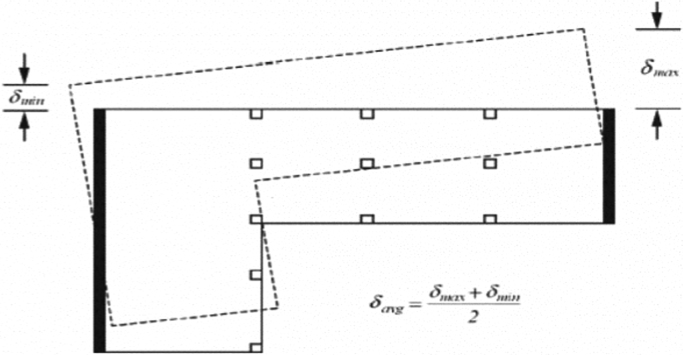
Extreme and average displacement points related to torsion [12].

Numerous studies have investigated various scenarios of torsional irregularity that causes structural collapse and vulnerability [9], [10], [11]. Earthquake field investigations have confirmed that irregular building structures suffer more damage than the regular ones. With regard to torsional irregularities, most of the seismic codes such as UBC97 and ASCE7, IBC09 have the same provisions and the same principles. According to ASCE7, the following two equations Eqs. (7) and (8) are used to check the validity and the existences of torsion on the building. Fig. 17 describes the extreme and average displacement points.

Torsional irregularity exists if:

(7) $$\frac{S\max}{\mathrm{Saverage}}>1.2$$

Extreme torsional irregularity exists if:

(8) $$\frac{S\max}{\mathrm{Saverage}}>1.4$$

### 2.6 Non-linear static analysis

**Fig. 18:**
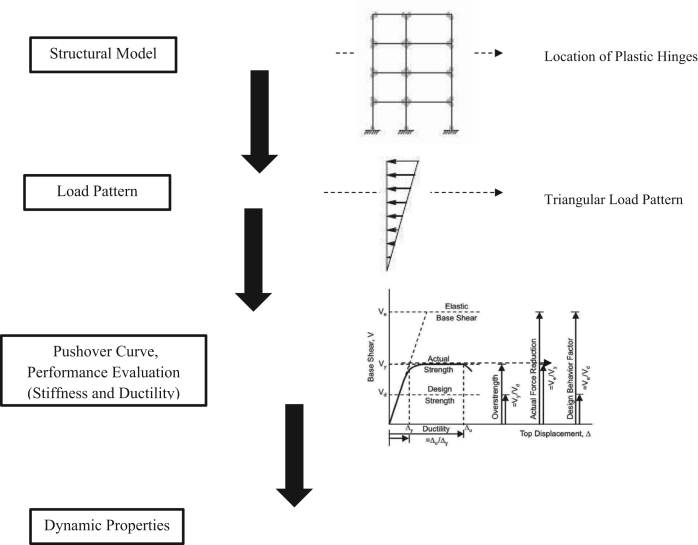
General flowchart for non-linear static analysis procedure.

Pushover analysis can be performed using ETABS software. It is a non-linear capacity estimation tool and popularly known as capacity analysis. In this case, capacity is the complex function representative of stiffness (*K*), ductility (*μ*) and deformation (δ) based on the material properties and building system. Many researchers use the pushover analysis as a tool in seismic assessment of the retrofitting techniques [13], [14], [15] with reference to FEMA273 [16]. In this article, the pushover analysis was done to check the performance of the existing building in the X-direction. The structural elements are modelled with concentrated plastic hinges at the column and beam faces, where the beams only had moment (M3) hinges, and the columns had an axial load and biaxial moment (PMM) hinges. These types of hinges are known as material inelasticity. The capacity curve for the three structural models describes the variation of base shear forces versus roof displacement (V vs. δ), where the base shear forces and the displacements were used as the control measuring parameters to obtain the stiffness and the ductility of each model as shown in Fig. 18. Eqs. (9) and (10) were used to find the ductility and stiffness values for each model.

(9) K=Vy/Δy

(10) μc=Δu/Δy
