# Supplementary material for: Dataset on the dynamic structural parameters of an old existing building exposed to earthquake loading before and after strengthening techniques – A case study, Lebanon
Source: Data Brief. 2018 Nov 20;21:2262–83. doi: 10.1016/j.dib.2018.11.077 (PMC6279946; doi:10.1016/j.dib.2018.11.077)
Supplement: Supplementary file 1 — Supplementary material [file mmc1.pdf]

Conflict of Interest and Authorship Conformation Form

Manuscript title: Dataset on the Dynamic Structural Parameters of an Old Existing Building Exposed to Earthquake Loading Before and After Strengthening Techniques- A case study, Lebanon.

Affiliations: [1]School of Civil Engineering, Universiti Sains Malaysia, Engineering Campus, 14300 Nibong Tebal, Penang, Malaysia

Journal: Data in Brief

Please check the following as appropriate:

- ○ All authors have participated in (a) conception and design, or analysis and interpretation of the data; (b) drafting the article or revising it critically for important intellectual content; and (c) approval of the final version.
- ○ This manuscript has not been submitted to, nor is under review at, another journal or other publishing venue.

This form is signed by all the authors to indicate the agreement of the above information is true and correct:

**Author's name**	**Author's signature**	**Date**
Moustafa Moffed Kassem	Moustafa Kassem	19th Sept 2018
Fadzli Mohamed Nazri	Fadzli Nazri	19th Sept 2018
